# Identification of effective screening strategies for cardiovascular disease prevention in a developing country: using cardiovascular risk-estimation and risk-reduction tools for policy recommendations

**DOI:** 10.1186/1471-2261-13-10

**Published:** 2013-02-25

**Authors:** Sharmini Selvarajah, Jamaiyah Haniff, Gurpreet Kaur, Tee Guat Hiong, Adam Bujang, Kee Chee Cheong, Michiel L Bots

**Affiliations:** 1 Clinical Research Centre, Ministry of Health Malaysia, Kuala Lumpur, Malaysia; 2 Julius Center for Health Sciences and Primary Care, University Medical Center Utrecht, Utrecht, The Netherlands; 3 Julius Centre University of Malaya, Kuala Lumpur, Malaysia; 4 Institute for Public Health, Ministry of Health Malaysia, Kuala Lumpur, Malaysia; 5 Institute for Medical Research, Ministry of Health Malaysia, Kuala Lumpur, Malaysia

**Keywords:** Cardiovascular risk, Cardiovascular disease, Policy, Screening

## Abstract

**Background:**

Recent increases in cardiovascular risk-factor prevalences have led to new national policy recommendations of universal screening for primary prevention of cardiovascular disease in Malaysia. This study assessed whether the current national policy recommendation of universal screening was optimal, by comparing the effectiveness and impact of various cardiovascular screening strategies.

**Methods:**

Data from a national population based survey of 24 270 participants aged 30 to 74 was used. Five screening strategies were modelled for the overall population and by gender; universal and targeted screening (four age cut-off points). Screening strategies were assessed based on the ability to detect high cardiovascular risk populations (effectiveness), incremental effectiveness, impact on cardiovascular event prevention and cost of screening.

**Results:**

26.7% (95% confidence limits 25.7, 27.7) were at high cardiovascular risk, men 34.7% (33.6, 35.8) and women 18.9% (17.8, 20). Universal screening identified all those at high-risk and resulted in one high-risk individual detected for every 3.7 people screened, with an estimated cost of USD60. However, universal screening resulted in screening an additional 7169 persons, with an incremental cost of USD115,033 for detection of one additional high-risk individual in comparison to targeted screening of those aged ≥35 years. The cost, incremental cost and impact of detection of high-risk individuals were more for women than men for all screening strategies. The impact of screening women aged ≥45 years was similar to universal screening in men.

**Conclusions:**

Targeted gender- and age-specific screening strategies would ensure more optimal utilisation of scarce resources compared to the current policy recommendations of universal screening.

## Background

Malaysia is one of the many developing countries in the world that has undergone epidemiologic and demographic transition. Recent national health reports showed a rising prevalence of several risk factors [1] and worrying clustering of cardiovascular risk factors [2]. However, information on risk factor prevalence alone is insufficient to provide adequate knowledge on the risk of future cardiovascular events. It is well known that a constellation of low to moderately elevated risk factors can confer a higher cardiovascular risk in an individual than just one highly elevated risk factor [3,4]. For example, a 45 year old male smoker, non diabetic with a total cholesterol level (TC) of 5.4 mmol/l, systolic blood pressure (SBP) of 150 mmHg and a HDL cholesterol level of 1.2 mmol/l has an overall 10-year cardiovascular disease risk of 17% compared to 8.9% of a 50 year old non smoker, non diabetic who has a SBP of 180 mmHg, total cholesterol of 4.3 mmol/l and HDL of 1.9 mmol/l (using the Framingham Risk Score). Therefore, cardiovascular risk estimation is an important component of estimating the overall effects of risk factors.

Recently, the Ministry of Health, Malaysia developed a national strategic plan to tackle the burgeoning increase in cardiovascular risk factors and disease. Among the various strategies and key activities planned are screening strategies to identify individuals at high cardiovascular risk to institute early clinical management. The two proposed strategies are: 1) to start community based risk factor screening (universal screening) and 2) to make policy and regulation changes to include compulsory screening for all employees aged 40 and above [5].

However, before the implementation of national policies, the most effective screening strategy should be identified. In this study, we hope to answer three questions; 1) What is the distribution of overall cardiovascular risk in Malaysia? 2) What are the more effective screening strategies to identify high-risk populations? and 3) What are the impact (numbers of cardiovascular events prevented) and estimated costs for these strategies?

## Methods

### Study population

This study used data from the National Health and Morbidity Survey (NHMS III) conducted in 2006. The NHMS is a national population based survey held every ten years, that assesses various aspects of health care, including burden of disease, health care utilisation and costs. The NHMS III used a two-stage stratified random sampling strategy proportionate to the population size of Malaysia. All data were collected via a face-to-face interview using a bi-lingual (Malay language and English) pre-coded questionnaire. The NHMS III was funded by the Ministry of Health Malaysia and ethics approval was obtained from the Medical Research and Ethics Committee, Ministry of Health Malaysia. Written informed consent was obtained from all participants prior to the interview and examinations. Details of this survey have been published previously [1]. Briefly, blood pressure was measured using the average of two readings of systolic and diastolic blood pressure taken at rest, 15 minutes apart. Blood glucose levels were measured after an overnight fast. Height was measured without shoes to the nearest 0.1 centimetre and body weight was measured in light clothing without shoes to the nearest 0.1 kilogramme.

Survey participants aged 30 to 74 years were selected for this study.

### Overall cardiovascular risk

Overall cardiovascular risk was estimated using the Framingham Risk Score (FRS) for general cardiovascular disease (10-year risk) [6]. Events of this risk score are coronary death, myocardial infarction, coronary insufficiency, angina, ischaemic stroke, haemorrhagic stroke, transient ischaemic attack, peripheral artery disease and heart failure. The FRS used a simple office-based non-laboratory set of variables. We used the formula with body mass index (BMI) as a substitute for total and high-density lipoprotein (HDL) cholesterol levels, because in the NHMS III, HDL cholesterol levels were not measured. The variables were logarithm of age, logarithm of BMI, logarithm of systolic blood pressure (SBP) (with different regression coefficients for treated or untreated high blood pressure), smoking and diabetes mellitus (website: http://www.framinghamheartstudy.org/risk/gencardio.html).

An example is given below: The 10-year risk of cardiovascular disease for men who were not treated for hypertension was calculated as 1–0.88431^exp((3.11296*logage) + (0.79277*logBMI) + (1.85508*logUntreatedSBP) + (0.70953*smoking) + (0.53160*diabetes) – 23.9388).^

#### Framingham risk definitions

High risk individuals were defined as those whose 10-year risk of cardiovascular disease was more or equal to 20%. Those at intermediate risk were between 10 to 20% and low risk was less than 10%.

### Statistical analyses

A complex survey analysis weighted for non-response, as well as population age and sex demographics, was used to produce correct estimations for the Malaysian population. Prevalences, screening coverage and detection rates of populations at high cardiovascular risk were estimated.

Prevalence estimates for demographics and cardiovascular risk factors were given by the Framingham risk categories, as well as overall. Variance was estimated using the Taylor linearization method [7]. Group differences between risk categories for continuous variables were estimated using an adjusted Wald test (*F* statistic). Differences between the risk categories for categorical variables were tested using Pearson’s chi square test, adjusted for design effect (*F* statistic).

For all analyses, p values less than 0.05 were considered statistically significant. Analyses were performed using Stata Statistical Software: Release 11.0 (College Station, TX: Stata Corporation LP).

#### Simulated screening strategies

For the purpose of this study, only the universal (community-based) screening policy recommendation was assessed, because this strategy will be funded by the government, and it encompasses the entire population. The other screening strategies chosen for simulation in this study were based on incremental five year age cut-offs. Stratification by gender was included to determine if gender-specific screening strategies were required. The coverage, effectiveness and impact of screening strategies were simulated for:

1. Universal screening (aged 30 and above)

2. Those aged 35 and above

3. Those aged 40 and above

4. Those aged 45 and above

5. Those aged 50 and above

#### Effectiveness

Effectiveness was assessed as the ability of a screening strategy to identify individuals of high cardiovascular risk as classified by the FRS. Comparisons of effectiveness were determined using the numbers needed to screen (NNS) to detect one high-risk individual. Incremental effectiveness was determined as the additional number of individuals needed to be screened to detect one high-risk individual. Strategies were compared with a lower age cut-off for screening eligibility.

#### Impact

The impact of each screening strategy was assessed by the NNS to prevent one cardiovascular event among individuals at high risk. The number of cardiovascular events prevented was determined using the following formula [8]:

Number of cardiovascular events prevented = N *x* Cardiovascular disease rate *x* (1-((1- pd *x* pu *x* pc *x* RRR)_int 1_*x* -(1- pd *x* pu *x* pc *x* RRR)_int 2_*x* …*x* -((1- pd *x* pu *x* pc *x* RRR)_int n_)

Where,

•N = number of high-risk people in respective screening strategy

•Cardiovascular disease rate = average FRS score for respective screening strategy

•pd = proportion of high-risk people with disease/ risk factor requiring intervention

•pu = proportion of high-risk people with disease/ risk factor requiring intervention that take up the intervention

•pc = proportion of adequacy of control /adherence to intervention

•RRR = relative risk reduction achieved with intervention [9-12]

The interventions that were assessed in the simulation models were antihypertensive, lipid lowering and glucose lowering drugs, and smoking cessation therapies.

##### Sensitivity analyses

We carried out sensitivity analyses to account for uncertainties in the parameters chosen for measuring the impact of the respective screening strategies. The parameters were for uptake of treatment, adherence to treatment and the relative risk reductions for those adhering to treatment. The uptake of treatment was calculated as a 30% reduction from the actual uptake seen in the NHMS III. Values for adherence to treatment and relative risk reductions were obtained from the lower limits of their 95% confidence intervals [1,9-13].

Table 1 depicts the percentage of individuals with a cardiovascular risk factor who decide to accept treatment, the percentage adhering to treatment and the relative risk reduction for those adhering to treatment, and the respective values chosen for sensitivity analyses.

**Table 1:** Uptake and adherence to treatment, and relative risk reductions for cardiovascular interventions

**Therapy/ Intervention**	Percentage of uptake *	Percentage of adherence*	**RRR ‡**
Antihypertensives	87.5	26.3	0.22
Lipid lowering drugs	44.1	69	0.22
Hypoglycaemic agents	85.8	29.3†	0.1
Smoking cessation	70.6	9.30	0.36
*Sensitivity analyses*			
Antihypertensives	57.5	24.8	0.17
Lipid lowering drugs	14.1	65.3	0.18
Hypoglycaemic agents	55.8	17.1	0.02
Smoking cessation	40.6	8.9	0.29

* from the NHMS III (1).

† from the diabetes registry Malaysia (13).

‡ from meta-analysis on effects on interventions on CVD events (5–8).

*RRR* relative risk reduction.

#### Cost

Cost estimations for each screening strategy were calculated using the Malaysian Medical Association’s Schedule of Fees [14]. The recommended fee for screening is Malaysian Ringgit (MYR) 50.00 (about USD16.00).

##### Assumptions

Those who do not adhere to therapies have the same 10-year risk of cardiovascular disease as those untreated. All interventions are independent of each other and there are no additive nor multiplicative effects.

## Results

There were 24 270 participants from the NHMS III survey between the ages 30 to 74 years. Women made up 55.2% of the population (13 393 participants).

### Distribution of overall cardiovascular risk

26.7% (95% confidence limits 25.7, 27.7) were in the high risk category, 20.3% (19.8, 20.9) were at intermediate risk and 53% (51.8, 54.1) were in the low risk category (Table 2). Among those in the low risk category, a quarter had hypertension and almost 40% were centrally obese.

**Table 2:** Characteristics of study participants by their overall cardiovascular risk

	**Overall**	**Low risk**	**Intermediate risk**	**High risk**	**p value**
**Variables**					
Age	49.4 (0.01)	48.4 (0.03)	49.7 (0.03)	52.9 (0.13)	
Male sex	49.6	40	55	64.3	<0.001
Race					0.008
Malay	48.4	47.2	48.3	50.9	
Chinese	29.6	28.3	30.3	31.5	
Asian Indian	7.8	7.9	8.2	7.4	
Others	14.2	16.6	13.2	10.1	
Residence					0.007
Urban	60.8	64.3	58	55.8	
Rural	39.2	35.7	42	44.2	
Education (Years of schooling)					<0.001
Tertiary (≥ 13 years)	7.8	11.1	5.8	2.8	
Secondary (7–12 years)	41.7	54.3	33.7	22.9	
Primary (≤6 years)	35.4	26.4	40.9	49	
Household income					<0.001
<RM2000	62.4	57.1	64.4	71.4	
RM2000-3999	23.9	26.3	23.1	19.8	
≥RM4000	13.7	16.6	12.5	8.8	
Prevalences of CV risk factors					
Smoking	22.2 (20.3, 24.3)	16.6 (14.8, 18.5)	25.9 (23.8, 28.0)	30.7 (27.7, 33.8)	<0.001
Central obesity	44.6 (42.6, 46.5)	38.4 (36.7, 40.2)	48.5 (45, 51.9)	53.8 (51, 56.5)	<0.001
Hypertension	50.1 (48.1, 52.2)	26.1 (24.4, 28.1)	64.3 (62.2, 66.5)	87 (85.9, 88)	<0.001
Hypercholesterolemia	30.3 (27.7, 33)	23.2 (21.0, 25.5)	35.9 (32.6, 39.3)	40.1 (36.8, 43.6)	<0.001
Diabetes	15.2 (13.8, 16.7)	4 (3.4, 4.6)	15.4 (13.7, 17.3)	37.3 (33.7, 41)	<0.001

Data are % for categorical variables, mean (se) for continuous variables & prevalence (95%CI) for risk factors.

*CV* cardiovascular.

Smoking; current smokers who smoked ≥100 cigarettes in their lifetime, and smoked daily or for some days in.

The previous month.

Central obesity; men, 90 cm and women, 80 cm [25].

Hypertension; SBP ≥ 140 mmHg and/or DBP ≥ 90 mmHg [26], or use of anti-hypertensive medication.

Hypercholesterolemia; total cholesterol level ≥ 5.2 mmol/l [27] or use of lipid lowering drugs.

Diabetes; fasting glucose ≥ 6.1 mmol/l [28] or self reported to be diabetic.

**Figure 1:**
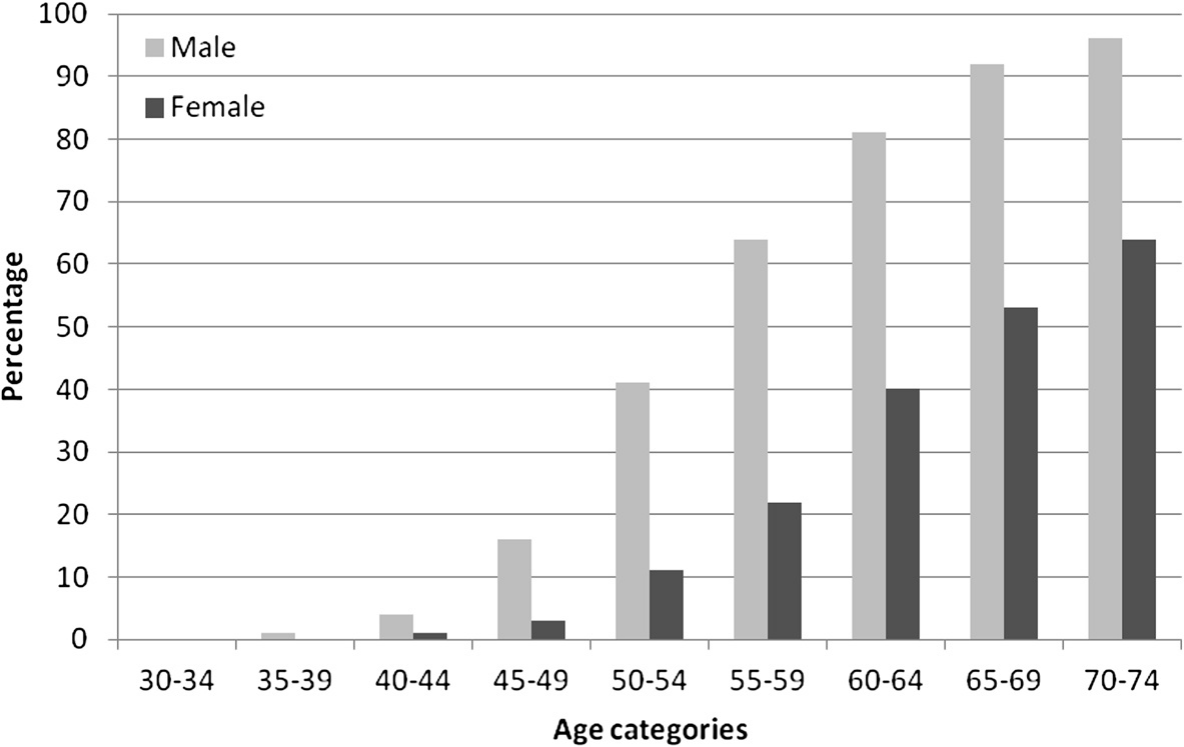
Percentage of males and females with high overall cardiovascular risk (> = 20% ten year risk) by age categories.

**Figure 2:**
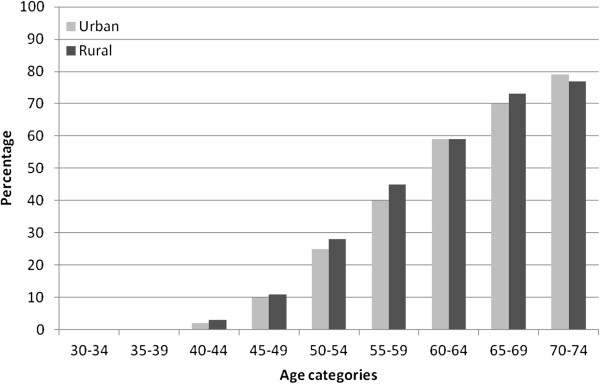
Percentage of urban and rural populations with high overall cardiovascular risk (> = 20% ten year risk) by age categories.

Overall, 34.7% (33.6, 35.8) men and 18.9% (17.8, 20) of women (p = 0.0001).were considered at high risk. For every age group, there were far more men at high risk of cardiovascular disease (Figure 1). The prevalence of high risk was similar in urban and rural areas (Figure 2).

### Coverage and detection of populations at high cardiovascular risk

As the cut-off age for screening strategies reduced, more of the general population were eligible for screening (Table 3). However, despite the increase in coverage of 53.3% from the cut-off age of ≥50 to universal screening, the coverage of high risk populations only increased by 5.6%. Aside from this, the high risk individuals detected formed smaller proportions of the screened population as the screened populations got larger.

**Table 3:** Coverage and detection of high cardiovascular risk populations for various screening strategies

	**Targeted screening**				**Universal**
	**≥ age 50**	**≥ age 45**	**≥ age 40**	**≥ age 35**	**screening**
**Overall**					
Coverage of population	46.72	58.06	70.13	84.47	100.00
Coverage of high risk population	94.39	98.71	99.82	100.00	100.00
% of high risk individuals - among those screened	53.97	45.42	38.02	31.62	26.71
NNS to detect 1 high risk individual (no.)	1.85	2.20	2.63	3.16	3.74
**Males**					
Coverage of population	44.36	56.32	68.82	83.95	100.00
Coverage of high risk population	92.60	98.22	99.75	100.00	100.00
% of high risk individuals - among those screened	72.41	60.50	50.28	41.32	34.69
NNS to detect 1 high risk individual (no.)	1.38	1.65	1.99	2.42	2.88
**Females**					
Coverage of population	49.03	59.76	71.41	84.98	100.00
Coverage of high risk population	97.61	99.58	99.99	100.00	100.00
% of high risk individuals - among those screened	37.59	31.47	26.43	22.22	18.88
NNS to detect 1 high risk individual (no.)	2.66	3.18	3.78	4.50	5.30

All data are in percentage (%) unless otherwise stated.

*NNS* numbers needed to screen.

### Effectiveness of screening strategies

The NNS to detect one high-risk individual increased as the cut-off age for screening was reduced. With universal screening, one high-risk individual was detected for every four people screened. Whereas, when only those over age 50 years were screened, one high-risk individual was detected for every two persons screened (Table 3). Furthermore, the NNS for men with universal screening (2.88) was far smaller than the NNS for women (5.30).

### Incremental effectiveness of screening strategies

As the screening population was extended (younger ages were included), the additional coverage of high-risk populations decreased, the percentage of additional high-risk individuals detected reduced and the additional number of individuals needed to be screened to detect one high-risk individual increased tremendously (Table 4). These findings were similar for men and women.

**Table 4:** Incremental coverage and detection of high cardiovascular risk populations for various screening strategies

	**Targeted screening**				**Universal**
	**≥ age 50†**	**≥ age 45**	**≥ age 40**	**≥ age 35**	**screening**
Strategies implemented incrementally*					
Additional % of population screened	-	24.27	20.79	20.45	18.39
Additional coverage of high risk population screened	-	4.32	1.11	0.18	0.00
% of additional high risk individuals detected	-	4.59	1.11	0.17	0.01
Additional NNS to detect 1 high risk individual	-	9.81	41.20	309.67	7168.90
**Males**					
Additional % of population screened	-	26.96	22.20	21.99	19.12
Additional coverage of high risk population screened	-	5.62	1.53	0.25	0.00
% of additional high risk individuals detected	-	6.08	1.55	0.25	0.01
Additional NNS to detect 1 high risk individual	-	6.12	23.61	176.52	5179.06
**Females**					
Additional % of population screened	-	21.88	19.49	19.00	17.67
Additional coverage of high risk population screened	-	1.97	0.41	0.01	0.00
% of additional high risk individuals detected	-	2.04	0.36	0.05	0.01
Additional NNS to detect 1 high risk individual	-	28.53	173.39	1525.92	5876.37

* reference screening strategy is directly to the left of screening strategy assessed.

† first reference group (for ages ≥ 45).

NNS numbers needed to screen.

### Impact

With universal screening, 147 people have to be screened and treated for 10 years to prevent one cardiovascular event (Table 5). For men, the NNS and treat for 10 years, to prevent a single cardiovascular event was lower than women for all screening strategies. These values increased by up to 2.6 – 2.8 times when accounted for uncertainties in the parameters.

**Table 5:** Cost, incremental cost and impact of screening strategies

	**Targeted screening**				**Universal**
	**≥ age 50†**	**≥ age 45**	**≥ age 40**	**≥ age 35**	**screening**
**Overall**					
Estimated cost to detect 1 high risk individual, MYR (USD)	92.64 (29.73)	110.08 (35.33)	131.51 (42.20)	158.13 (50.75)	187.2 (60.08)
Incremental cost per additional high risk individual detected, MYR	-	490.27	2060.12	15483.29	358444.85
(USD)	-	(157.34)	(661.14)	(4968.96)	(115033.65)
NNS to prevent one CV event	62.24	76.14	96.64	123.95	146.73
NNS to prevent one CV event (sensitivity analyses)	168.47	206.07	261.57	335.48	397.15
**Males**					
Estimated cost to detect 1 high risk individual, MYR (USD)	69.05 (22.16)	82.64 (26.52)	99.44 (31.91)	121.01 (38.84)	144.13 (46.25)
Incremental cost per additional high risk individual detected, MYR	-	306.21	1180.58	8825.99	258952.77
(USD)	-	(98.27)	(378.88)	(2832.47)	(83104.23)
NNS to prevent one CV event	45.33	57.27	72.96	94.33	112.36
NNS to prevent one CV event (sensitivity analyses)	120.13	151.77	193.36	249.97	297.77
**Females**					
Estimated cost to detect 1 high risk individual, MYR (USD)	133.01 (42.69)	158.88 (50.99)	189.18 (60.71)	225.02 (72.21)	264.83 (84.99)
Incremental cost per additional high risk individual detected, MYR	-	1426.50	8669.32	76295.96	293818.47
(USD)	-	(457.8)	(2782.19)	(24485.23)	(94293.47)
NNS to prevent one CV event	85.72	105.59	129.78	165.01	194.20
NNS to prevent one CV event (sensitivity analyses)	240.97	394.25	364.83	463.95	545.95

*CV* cardiovascular, *NNS* numbers needed to screen.

*MYR* Malaysian Ringgit, *USD* US Dollar. 1.00 USD = 3.11600 MYR.

* for incremental costs, reference screening strategy is directly to the left of screening strategy assessed.

† first reference group (for ages ≥ 45).

### Cost of screening strategies

The estimated cost of detecting each high-risk individual increased as the screening strategy progressively encompassed younger populations (Table 5). When comparing the screening strategies of the universal screening and those aged ≥50 years, the cost for detecting a single high-risk individual slightly more than doubled. The cost of detecting high-risk individuals among men were almost half that of women, for all screening strategies.

### Incremental costs

The cost of detecting one additional high-risk individual increased exponentially as the targeted screening population coverage increased incrementally (Table 5). As younger and younger individuals were screened, the additional cost for detecting one high-risk individual differed significantly among the sexes. For those aged ≥45 years, the incremental cost for detecting an additional high-risk individual among women was 4.7 times that of men. Once the eligibility age of screening reduced to ≥ 35 years, the incremental cost among women was 8.6 times that of men.

## Discussion

Our study shows that a targeted cardiovascular risk screening strategy would be better than the policy recommendation of screening for all ages at a community level (universal screening). Defining an age eligibility criteria would be a more cost effective method of identifying high-risk individuals. In addition, our study highlights the need for different screening strategies for men and women due to a significant difference in their overall cardiovascular risk.

The findings of our study have important implications for policy makers in the prevention and management of cardiovascular disease. Firstly, high rates of cardiovascular risk factors in the country, do not necessarily translate into high overall cardiovascular risk. A previous study on cardiovascular risk factors showed a very high prevalence of hypertension (38%), diabetes (11%), hypercholesterolemia (24%), central obesity (37%) and it’s clustering (33%) [2]. This was more pronounced in women. Our study showed that overall cardiovascular risk was more severe in men than women, for all ages. Therefore, having identical screening strategies for both sexes may not be necessary or cost-effective.

Secondly, there are various factors which help determine the optimal screening strategy to be recommended; the numbers needed to screen to detect one high-risk individual, its cost and number of cardiovascular events prevented. Our results show that universal screening would cost twice as much as screening those aged ≥50 but detect high-risk individuals at half the rate. Aside from that, universal screening would only detect an additionally very small percentage of high-risk individuals.

Thirdly, comparing screening strategies by estimating the incremental cost and effectiveness provides a clearer picture of how much more is paid to identify an additional high-risk individual. By limiting the age for screening to just ≥35 years compared to the general community, the incremental cost spent for detecting one additional high-risk individual can be brought down significantly. Aside from this, the discrepancies in cost are substantial between the sexes. Choosing the optimal screening strategy will depend on the amount policy makers and financiers are willing to pay for each additional high-risk individual detected. Finally, the impact of these strategies clearly show that universal screening only marginally reduces the numbers of cardiovascular events prevented over ten years, when compared to screening those aged 50 and above.

Ideally, the decision to recommend a cardiovascular screening strategy should depend on two factors. First, the ability of the screening strategy to detect the highest proportion of high-risk individuals at an acceptable cost, and second, the ability of healthcare facilities to manage the treatment of these individuals from a financial and human resource perspective. Policy makers and programme planners will have to take these factors into account when deciding the recommended screening strategy for the country. This is especially important for Malaysia where up to 64.5% of the population seek health care from public facilities funded by the government [1]. This study highlighted that the high incremental costs and very low impact for universal screening may not be justifiable for implementation.

From a public health perspective, this study illustrates that developing countries without available information on, or the resources to obtain information on long term risk of cardiovascular disease and outcomes, can use existing cardiovascular risk scores and global risk-reduction estimates to make informed decisions. While these estimated may not be 100% accurate, they provide a clear picture on the impact of various screening strategies based on observed risk-factor prevalences in the country.

There have been very few studies which have examined the effectiveness and impact of screening strategies for the prevention of cardiovascular diseases using real population data. Chamnan et al. [8] assessed the potential impact of various screening strategies in the United Kingdom using data from a single county. They too found that limiting screening to those older than 50 years, or using routine general practice data already available to pre-stratify high-risk individuals were more (cost) effective than screening the general population. For Scotland, Lawson et al. found that targeting individuals with a family history of premature cardiovascular disease was the most cost-effective measure [15]. However, their assessment of cost-effectiveness only took into account the cost of screening and identification of high-risk individuals. They did not account for the impact of screening. Our study used data from a nationwide population-based survey and took into consideration the observed treatment uptake and adherence rates. Our study also accounted for the impact of treatment on high-risk individuals for the various screening strategies.

Our study is not without limitations. The Framingham Risk Score has not been validated in our multi-ethnic population. Therefore, it’s accuracy in prognostication of risk is unclear. However, it has been shown to accurately risk stratify but overestimate cardiovascular risk in some European, Australian and Middle Eastern populations [16-18]. An earlier version of the FRS algorithm [19] had been validated in a Chinese population. For the Chinese population, it too accurately risk stratifies but overestimates the coronary heart disease risk [20]. If the FRS similarly overestimates cardiovascular risk in Asian populations, the findings of this study are even more important, because for similar risk scores, less cardiovascular events occur in Asians. Thus, screening strategies can be recommended for those with higher Framingham risk scores (eg. 30% and above), or for older age groups because they have higher proportions of high-risk individuals. For women, a higher risk score cut-off than men will be warranted. Aside from this, with lower actual cardiovascular rates, the impact of these screening strategies would be even more reduced for every estimated cardiovascular risk. The numbers needed to screen to prevent a single cardiovascular event would increase substantially. This would be more so for women, with lower age cut-off points, and would be worst with universal screening.

Similarly, relative risk reductions for treatment of cardiovascular risk factors have not been assessed in our population. Nevertheless, research has confirmed that risk reduction estimates have been found to be consistent across populations around the world [21]. In this study, the relative risk reductions were for cardiovascular disease outcomes except for smoking, which was related to mortality.

In our study, the relative risk reduction for each intervention was assumed to be independent of each other. Synergistic effects of multiple drug and lifestyle interventions may be present and most likely will have different impacts for the various screening strategies. However, this may also be true for side effects and complications of treatment, thus the true impact of any treatment strategy may not be so easily determined.

We did not account for cardiovascular screening uptake in this study. In other populations, screening uptake can be as low as 32% [22]. In Malaysia, there has been one published paper which described cardiovascular screening uptake in a semi-rural community in 1993 [23]. The response rate to screening invitation was 56%. However, it ranged up to 90% depending on the village invited. This high variability in screening uptake suggests that the modelled strategies in this study will have differing impact and effectiveness, depending on the screening acceptance rates of the respective communities as well as the cardiovascular risk distribution among respondents. As we were not able to estimate the variability of cardiovascular risk distribution in participants for various rates of screening uptake, we chose to model a 100% uptake for universal screening, with a cardiovascular risk distribution of the survey participants of the NHMS III. In the NHMS III, the response rate was 94.6% [1]. This method represents the best case scenario for the Malaysian context. In the study by Chin et al., non-responders to the cardiovascular screening invitation were of a similar age-and-gender distribution as responders [23]. Aside from this, in a Japanese study of non-participation to screening and mortality risks, they showed that the lack of participation to screening was not associated with an increased risk of cardiovascular mortality for men [24].

## Conclusion

Policy recommendations for general cardiovascular screening should be gender-specific with different age group targets. This is to ensure optimal utilisation of scarce resources for the identification of high-risk individuals in the prevention of cardiovascular disease in Malaysia.

## Competing interests

The authors declare that they have no competing interests.

## Authors’ contribution

Authors JH, GK and TGH were involved in the conception and design of the study, acquisition of data as well as drafting of the manuscript. Author SS was involved in the conception, design, analysis and interpretation of the data as well as drafting the manuscript. Author AB and KCC were involved in the acquisition of data, and revising the manuscript critically for important intellectual content. Author MLB was involved in the analysis and interpretation of the data as well as revising the manuscript critically for important intellectual content. All the authors have read the final manuscript and have given their approval for it to be presented in its present form.

## Pre-publication history

The pre-publication history for this paper can be accessed here:

http://www.biomedcentral.com/1471-2261/13/10/prepub
